# Chimpanzees use social information to acquire a skill they fail to innovate

**DOI:** 10.1038/s41562-024-01836-5

**Published:** 2024-03-06

**Authors:** Edwin J. C. van Leeuwen, Sarah E. DeTroy, Daniel B. M. Haun, Josep Call

**Affiliations:** 1 Animal Behaviour and Cognition, Department of Biology, Utrecht University, Utrecht, the Netherlands; 2 Department of Comparative Cultural Psychology, Max Planck Institute for Evolutionary Anthropology, Leipzig, Germany; 3 Centre for Research and Conservation, Royal Zoological Society of Antwerp, Antwerp, Belgium; 4 School of Psychology & Neuroscience, University of St Andrews, St Andrews, UK

**Keywords:** Animal behaviour, Biological anthropology

## Abstract

Cumulative cultural evolution has been claimed to be a uniquely human phenomenon pivotal to the biological success of our species. One plausible condition for cumulative cultural evolution to emerge is individuals’ ability to use social learning to acquire know-how that they cannot easily innovate by themselves. It has been suggested that chimpanzees may be capable of such know-how social learning, but this assertion remains largely untested. Here we show that chimpanzees use social learning to acquire a skill that they failed to independently innovate. By teaching chimpanzees how to solve a sequential task (one chimpanzee in each of the two tested groups, *n* = 66) and using network-based diffusion analysis, we found that 14 naive chimpanzees learned to operate a puzzle box that they failed to operate during the preceding three months of exposure to all necessary materials. In conjunction, we present evidence for the hypothesis that social learning in chimpanzees is necessary and sufficient to acquire a new, complex skill after the initial innovation.

## Abstract (web summary)

Van Leeuwen and colleagues demonstrate that chimpanzees use social learning to acquire a skill they failed to innovate, supporting the hypothesis that social learning is necessary for acquiring complex skills after initial innovation.

## Main

Cumulative cultural evolution (CCE) is the process by which incremental accumulations of changes to a cultural trait lead to functional improvement in performance^1^ and is generally viewed as a hallmark of the human species^2–4^. CCE requires social learning, in which individuals acquire information about their environment by observing or interacting with their conspecifics or the products of their conspecifics’ actions^5,6^. While social learning has been invoked as an explanatory mechanism for large-scale between-group differences in behaviour in many non-human animal species^7–13^ (henceforth ‘animals’), a suite of recent work has challenged the idea that social learning in the specific variation of know-how copying is within the capacities of the great apes^14^. Specifically, this position in the animal culture debate has been coined the ‘zone of latent solutions’ (ZLS) hypothesis, and it states that apes, rather than copying know-how from each other, individually re-innovate behaviours that in conjunction become group-level traditions as an emergent property. Here, know-how copying is represented as the particular kind of social learning that is involved in acquiring knowledge on the production of a particular trait (for example, how to make a bow and arrow or how to dance the tango^15^)—as such, the ZLS hypothesis does not exclude other forms of social learning from the capacities of great apes (and other animals). Re-innovation is seen as the act of recreating a behaviour that already exists within the respective species’ repertoire by means of individual (and low-fidelity social) learning, thus without copying know-how from already proficient individuals^16^. The ZLS itself refers to an imagined space of know-how that members of a species can invent individually if basic conditions hold^17^. In support of their hypothesis, ZLS proponents point to studies in which captive apes without relevant prior knowledge invent behaviours that are widely regarded as requiring (cross-generational) know-how copying, such as nut cracking^18^ and algae scooping^16^ in chimpanzees and nettle feeding in gorillas^19^. The ZLS rationale is that if individual apes can invent the behaviour on their own, the most parsimonious assumption is that their counterparts in the wild have acquired the skill by individual re-innovation supported by social learning mechanisms other than know-how copying (for example, copying know-where or know-what)^16^. Furthermore, ZLS proponents refer to studies in which great apes failed to copy arbitrary human gestures^20,21^ as evidence for the apes’ inability to engage in know-how copying^14^. Taken together, the ZLS hypothesis posits that know-how copying is a uniquely human capacity that directly feeds into their unique cultural phenotype.

Recently, an encompassing review on comparative cultural cognition posited that high-fidelity transmission in the form of know-how copying and CCE is either already documented or at least within the range of possibilities for animals^22^. In this review^22^, a series of experimental studies in which naive animals (for example, birds and apes) selectively copied one of two possible foraging techniques following an initial innovator was put forward as evidence for know-how copying^23–25^. The argument goes that individual re-innovation of the foraging behaviour would lead to an equal distribution of usage frequency for both techniques, whereas only know-how copying could render the observed patterns of skewed frequencies^22,23^ (but see ref. ^26^ for a critique of this argument). Another recent study alluded to the presence of know-how copying in wild chimpanzees by identifying hitherto overlooked cultural complexity in their termite-fishing techniques. Both the number and combination of elements within the practice of termite fishing showed community specificity to the extent that both process-oriented imitation and cumulative cultural diversity could be inferred, according to the respective authors^8^ (also see ref. ^27^ for experimental indications of chimpanzees’ composite tool use and their social learning thereof). Moreover, a thought-provoking experimental study on the flying routes of homing pigeons challenged the very idea of know-how copying being essential for the ensuing of CCE. This study showed that pigeons increased the efficiency of their routes across several (cultural) generations in which they could iteratively benefit from input by newcomers in the absence of know-how copying^28^, which, according to the current gold standard, qualifies as CCE^1^.

The ZLS hypothesis, however, specifically focuses on know-how copying, which in its proponents’ view is the key to CCE as it enables the adoption of behaviours that are not already within the species’ repertoire. For instance, in the two-action methodology studies, both techniques (for example, poking and lifting a stick^24^) typically get invented by individuals in the control condition (that is, without social demonstrations)^29,30^, which leads the ZLS account to conclude that both behaviours are within the ZLS of chimpanzees and thus that know-how copying for such behaviours is not required^26^. Specifically, the ZLS account argues that in addition to individual learning, many low-fidelity types of social learning may be at play while learning the new behaviour (for example, know-where or know-what copying), but know-how copying need not be invoked to explain the selective diffusion of one over the other technique^14,31^. In this light, it has been suggested that naturally occurring behaviours that emerge and diffuse spontaneously provide a better argument for the existence of know-how copying in apes. For instance, chimpanzees have been observed to adopt unconventional postures and gaits of conspecifics^32,33^ and to put grass in their ears after observing one of their group members doing it^34^. These examples raise the question of how likely such phenomena can be explained as emergent properties of a series of individual re-innovations, especially given the relatively quick diffusion of these behaviours^35^. Nonetheless, these cases lack the empirical rigor to identify know-how copying beyond reasonable doubt^26,31^, which means that the jury is still out on the question of whether great apes—humans’ closest living evolutionary relatives^36^—merely re-innovate behaviours within their cultural lives, or whether they can use know-how copying to extract information from their environment, which could serve as important building blocks for an ensuing cumulative culture^37^.

A recently published experimental study on wild chimpanzees was conducted to target this question. In this study, all necessary materials for nut cracking were provisioned to a community of wild chimpanzees who were naive to the respective practice (while some of their neighbouring groups are known to engage in nut cracking regularly)^38^. After the chimpanzees were tested for more than a year at two experimental sites, in which roughly 100 visits were paid to the nut-cracking sites by roughly 20 individuals, the naive chimpanzees never managed to learn the nut-cracking skill^38^. Even though already-cracked nuts (the end-state condition) were provided to the chimpanzees in this study, they did not invent nut cracking. The authors concluded that low-fidelity social learning mechanisms, such as stimulus and local enhancement, did not suffice and that specifically know-how copying (that is, a high-fidelity social learning mechanism) was necessary for the chimpanzees to learn nut cracking. In consequence, according to the authors, nut cracking may best be seen as an outcome of cumulative culture^38^, which was hitherto considered unique to the human species^3^. Two issues remained outstanding: (1) the naive chimpanzees may not have been motivated to engage in nut cracking or eat the nuts, which arguably forms a prerequisite for learning a new skill^18,39^ (but see ref. ^40^), and (2) while the authors imply that know-how copying is within the range of capacities for chimpanzees, this implication itself remained empirically untested^38^.

A key question thus remains whether great apes can apply know-how copying to learn a behaviour they do not manage to re-innovate by themselves during a substantial amount of time. To address this question, we conducted a controlled behavioural experiment on skill acquisition in semi-wild chimpanzees at an African sanctuary (Chimfunshi Wildlife Orphanage Trust, Zambia). We tested whether chimpanzees (*n* = 66) could individually re-innovate a complex foraging skill by exposing them to an apparatus for which sequential actions were required to operate it successfully (that is, obtain a food reward). The required sequence was modelled after naturally occurring behaviour in which chimpanzees bring tools to foraging sites. For instance, when chimpanzees fish for termites, they go through a sequence involving collecting a probe (such as a suitable branch), opening a hole in the termite mound, inserting and extracting the probe, and collecting the termites^41,42^. The required sequence thus followed a vending-machine principle involving three main steps: (1) a wooden ball needed to be retrieved, (2) a drawer in the apparatus needed to be pulled out and kept protruded, and (3) the ball needed to be inserted into a cavity of the pulled-out drawer. Once the sequence had been successfully performed, the ‘loaded’ drawer had to be pushed back into the apparatus to trigger the release of a food reward in a designated compartment below the drawer. The task thus required a sequence of actions (collect ball, transport ball, pull drawer, keep drawer pulled out while inserting the ball, push drawer), thereby mirroring the sequential nature of various forms of chimpanzees’ tool-aided extractive foraging behaviours (for example, termite fishing^41^ and leaf spooning^43^). Moreover, the vending-machine principle minimized monopolization by high-ranking chimpanzees (that is, after the insertion of the ball and reward collection, there was nothing to be gained at the apparatus because a new ball was needed) and thus optimized observation opportunities and the number of chimpanzees that could attempt and solve the apparatus (for details, see the Supplementary Information).

First, we installed the apparatus in the chimpanzees’ enclosures (in two separate groups; Methods) and exposed the chimpanzees for prolonged periods to the setup with all necessary materials plentifully available. Second, after observing for three consecutive months that none of the chimpanzees invented the requisite action sequence, we selected one chimpanzee from each group to be trained as conspecific models for their group members. Finally, we re-ran the experiment in both groups with one skilful individual now present in each group.

Here we tested under controlled yet semi-natural conditions^44,45^ whether chimpanzees can learn a skill from conspecifics that proves difficult for them to acquire by themselves. The ZLS hypothesis predicts that chimpanzees will be unable to achieve this because they lack the capacity of know-how copying. In this case, the know-how resided in the sequential nature of the task, which included the selection, transportation and three-action manipulation of an item and the apparatus to release a food item. With this sequence, we aimed to simulate chimpanzees’ natural dynamics (for example, chimpanzees selecting tools to bring to and wield at termite fishing sites^29,42^) and substantially decrease the odds that the chimpanzees would innovate the behaviour by accident or coincidence. Our experiment consisted of two connected parts: (1) we tested whether naive chimpanzees who were exposed to all relevant materials could master the required skill by themselves within a prolonged period; and if not, subsequently (2) we tested whether naive chimpanzees could acquire the skill by observing a proficient (trained) conspecific model. Understanding whether chimpanzees possess the capacity to copy know-how from conspecifics may importantly contribute to tracing the evolutionary origins of human cumulative culture^1,3,22,46^.

## Results

### Part 1: the baseline

**Fig. 1:**
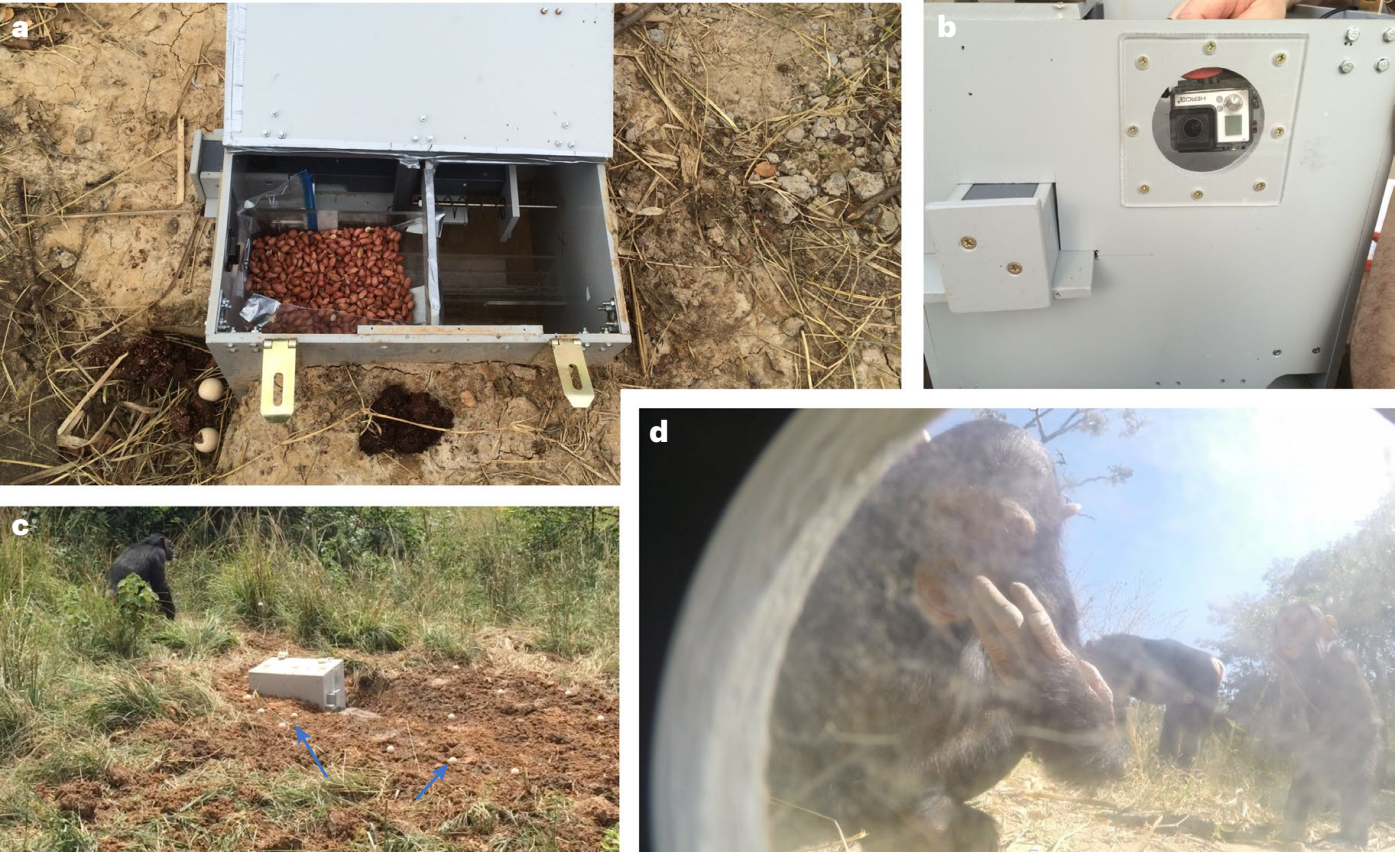
Experimental setup during the baseline phase. **a**–**d**, The apparatus (**a**,**b**) was introduced in the enclosures of Groups 1 and 2, after which the chimpanzees inspected and attempted to solve it (**c**,**d**). The apparatus worked with a drawer mechanism (**b**) that the chimpanzees needed to pull out to uncover the hole in the drawer, in which they needed to insert a wooden ball (Supplementary Video 1). If they were successful, they automatically received a food reward owing to the workings of an electric food-dispensing mechanism inside the apparatus. The blue arrows in **c** indicate the locations of balls needed for the drawer. Image **d** was extracted from the motion-sensitive camera that was built into the apparatus to capture any attempts made by the chimpanzees throughout the day and even during dusk and dawn. At no point during the baseline phase was a chimpanzee successful at operating the apparatus.

To test whether the required skill to operate the apparatus (Supplementary Video 1) would be innovated by the chimpanzees in the absence of inputted social information (that is, within their ZLS^14^), we installed one apparatus in each group and monitored the chimpanzees’ responses (Fig. 1). The apparatus was fully operational, which meant that upon the insertion of a wooden ball into the drawer mechanism, a food reward would be released automatically. Motion-sensitive cameras recorded bouts in which the chimpanzees manipulated the apparatus (for examples, see Supplementary Videos 2–6). We obtained 71 videos from one group in which several chimpanzees were attempting to retrieve food from the apparatus. However, due to data loss (theft at the university), we lack physical evidence of hundreds of attempts that were recorded in both groups (personal observations). Nonetheless, in conjunction, the videos and our observations showed that the chimpanzees were motivated to solve the apparatus and retrieve the food rewards (Supplementary Videos 2–6). Moreover, in each group, the chimpanzees once managed to break off the lid of the apparatus (during the first week of the baseline phase) and freely forage on the stored peanuts (Fig. 1a), which in our view attests to their interest and further sparked their motivation to solve the apparatus. Yet, across the entire baseline period of three months in each group, with all resources available to solve the apparatus (~75 balls close to the apparatus at all times; Methods and Supplementary Information), not a single chimpanzee out of the 66 residents was successful in operating the apparatus through the drawer mechanism once. We take this as an indication that individual learning, in combination with social cues provided by other naive group members (for example, local and stimulus enhancement^6^), did not suffice for the motivated chimpanzees to acquire the necessary skill in the allotted time.

### Part 2: social transmission

After training one chimpanzee in each group (a mid/high-ranking adult female; Methods) on the contingencies of the apparatus, we administered 39 two-hour sessions in each group during which the trained model started using the apparatus and thus functioned as a conspecific demonstrator for the rest of the group (Supplementary Fig. 1).

**Fig. 2:**
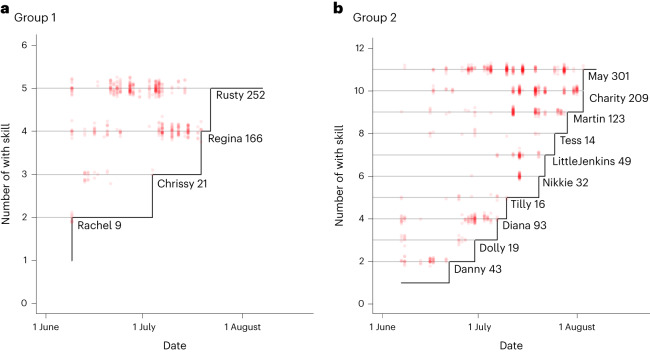
Observational records of the chimpanzees who mastered the skill. **a**,**b**, The red dots represent events in which individuals (*y* axis) observed a group member successfully operating the apparatus in Group 1 (**a**) and Group 2 (**b**). On the *x* axis, the experimental time is depicted. All chimpanzees who eventually mastered the skill had observed a successful token insertion and subsequent reward retrieval at least once (the number following each chimpanzee’s name represents the number of their observations prior to their first successful apparatus operation).

**Fig. 3:**
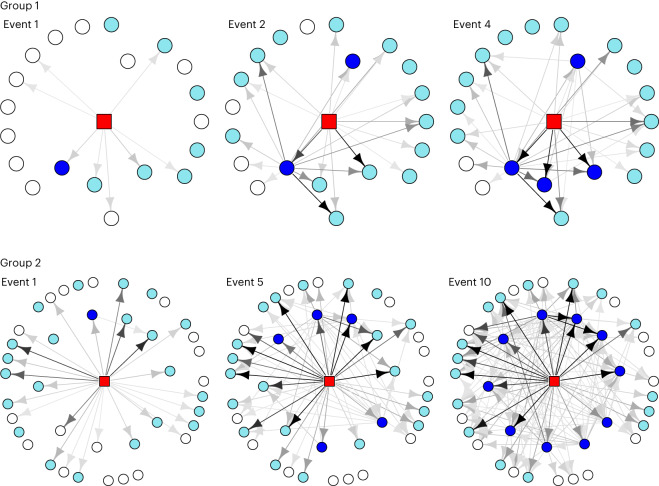
Social transmission of a new skill in two chimpanzee communities. The top row of panels refers to Group 1, the bottom row of panels to Group 2. Time goes from left to right; the event number refers to the number of naive (non-trained) chimpanzees having learned the skill. The trained demonstrator is shown as a red square, skilled individuals as dark-blue circles, attempting but not yet skilled individuals as turquoise circles, observing but not yet attempting individuals as white circles receiving arrows and and fully naive individuals as white circles without receiving arrows. The arrows go from observed to observer. The blackness of the arrows is proportional to the number of observations, with fully black representing 100 or more observations. Some chimpanzees attempted without having observed the solution, but all chimpanzees who learned the skill observed successful others at least once before learning. At the same time, there were many chimpanzees who frequently observed others but never learned the skill, which further attests to the difficulty of the skill in line with the pre-training baseline during which no chimpanzee was successful.

Across the two groups, there were 14 naive chimpanzees who at some point during the experimental sessions mastered the skill (Figs. 2 and 3). All these skill adopters had observed a model successfully solving the task at least nine times (Fig. 2), where ‘observations’ were scored when the focal was within 1.5 m of the model. Table 1 provides information on the chimpanzees’ observation efforts and subsequent skill mastery.

**Table 1:** Chimpanzees’ observation records and successful operations

Chimpanzee	Group	No. of observations	Acquired skill (0, no; 1, yes)	Total observation time (hours)	Rate of observation	No. of solves
BJ	1	205	0	71.38	2.87	0
Bob	1	13	0	71.38	0.18	0
Booboo	1	0	0	71.38	0	0
Brenda	1	56	0	71.38	0.78	0
Chrissy	1	21	1	28.99	0.72	333
Genny	1	24	0	71.38	0.34	0
Gerard	1	0	0	71.38	0	0
Girly	1	0	0	71.38	0	0
Gonzaga	1	99	0	71.38	1.39	0
Ilse	1	32	0	71.38	0.45	0
Ingrid	1	13	0	71.38	0.18	0
Innocentia	1	44	0	71.38	0.62	0
Irene	1	153	0	71.38	2.14	0
Pal	1	1	0	71.38	0.01	0
Rachel	1	9	1	2.32	3.88	926
Regina	1	166	1	45.77	3.63	1,048
Renate	1	406	0	71.38	5.69	0
Rita^a^	1	NA	NA	NA	NA	1,970
Rusty	1	252	1	49.17	5.12	1
Tara	1	3	0	71.38	0.04	0
Tobar	1	7	0	71.38	0.1	0
Carol	2	17	0	71.38	0.24	0
Charity	2	209	1	69.38	3.01	23
Claire	2	0	0	71.38	0	0
Coco	2	3	0	71.38	0.04	0
Daisey	2	75	0	71.38	1.05	0
Danny	2	43	1	71.38	9.85	3
David	2	0	0	71.38	0	0
Debbie	2	13	0	71.38	0.18	0
Diana	2	93	1	36.11	2.58	377
Dizzy	2	188	0	71.38	2.63	0
Dolly	2	19	1	29.2	0.65	1,381
Donna	2	25	0	71.38	0.35	0
Dora	2	33	0	71.38	0.46	0
Doug	2	0	0	71.38	0	0
Jacky	2	239	0	71.38	3.35	0
Jane	2	19	0	71.38	0.27	0
John	2	174	0	71.38	2.44	0
Jones	2	274	0	71.38	3.84	0
Judy	2	135	0	71.38	1.89	0
LittleJenkins	2	49	1	50.4	0.97	232
Maggie	2	289	0	71.38	4.05	0
Martin	2	123	1	54.63	1.85	1
Mary	2	59	0	71.38	0.83	0
Masya	2	13	0	71.38	0.18	0
Max	2	124	0	71.38	1.74	0
Maxine	2	31	0	71.38	0.43	0
May	2	301	1	69.38	4.47	3
Mikey	2	41	0	71.38	0.57	0
Misha	2	43	0	71.38	0.6	0
Moyo	2	39	0	71.38	0.55	0
Nikkie	2	32	1	49.15	0.65	213
Nina	2	8	0	71.38	0.11	0
Noel	2	14	0	71.38	0.2	0
Pan	2	0	0	71.38	0	0
Pippa^a^	2	NA	NA	NA	NA	3,453
Tess	2	14	1	54.78	0.33	179
Tilly	2	16	1	38.35	0.42	159
Trixie	2	11	0	71.38	0.15	0
Violet	2	38	0	71.38	0.53	0
Vis	2	25	0	71.38	0.35	0
Zsabu	2	9	0	71.38	0.13	0

^a^Trained individual, thus not taken into account for detecting social learning signatures. NA, not applicable.

The table shows the number of observations prior to the first solve for those that acquired the skill and prior to the final solve for those that did not; the time the chimpanzees had to make observations, which is the time until the first solve or the time until the final solve; the rate of observation (prior to the first solve for solvers); and the total number of solves for each individual.

To further identify whether the chimpanzees used social information to acquire the skill, we analysed the transmission data using network-based diffusion analysis (NBDA) in the R statistical environment v.3.6.1 (ref. ^47^) using the NBDA package v.0.8.3 (ref. ^48^). NBDA infers social transmission if the diffusion follows the connections of a social network representing opportunities to learn between members of each dyad^49,50^. In an NBDA, the strength of social transmission is estimated as the parameter *s*, defined as the rate of learning per unit connection with informed individuals (that is, an observation of an individual solving the task), relative to a baseline rate of asocial learning (here set to be the asocial learning rate for females of middle rank and average age). We used the order-of-acquisition variant of NBDA^50^, which takes as data the order in which individuals acquire the target behaviour (for more details, see the Supplementary Information).

The results of the NBDA support the hypothesis that chimpanzees used social learning to acquire the skill they had not been able to master via asocial learning alone (Supplementary Table 1). Specifically, the rate of social transmission was best predicted by the number of task solutions observed (support, 50.4%), followed by the number of individuals observed solving the task (support, 34.7%). One single observation of a task solution did not seem to be sufficient for a full social learning effect to occur (support, 13.5%). Models in which *s*_1_ = *s*_2_ were the best supported, suggesting that social transmission occurred in both groups and that there was no evidence of a difference in the magnitude of the social effect between groups. The absolute observation network with *s*_1_ = *s*_2_ received 65.3× more support than the group network, further supporting the hypothesis of social transmission following the pattern of observations within each group. Asocial learning received relatively little support at 0.6%; however, only four models were fitted, so we use the 95% confidence intervals (CIs) for *s* (see below) to quantify the strength of evidence against purely asocial learning (*s*_1_ = *s*_2_ = 0). Overall, given that all but the least supported model (Akaike weight, 5 × 10^−7^) estimated a lower 95% CI limit for *s* at >0, we conclude that social transmission of the task solution occurred (for more details, see Supplementary Information section 4).

Estimates of social effects were made conditional on the absolute observation network since this network received the most support. For a dynamic observation network, the *s* parameter estimates the increase in rate of solving per observation, relative to the baseline rate of asocial learning (set to a female of middle rank and age). Conditional on the best-fitting model, the 95% CI for *s* was 0.461–infinity, meaning the data provide a lower plausible limit on the size of s. This means that for every observation, chimpanzees’ learning rate increased at least 0.461× the asocial baseline rate: for example, a female of middle rank and average age who has observed ten solves would be expected to solve the task at least 1 + 10 × 0.461 = 5.61× faster than a comparable individual who has observed no individual solving the task. The 95% CI for *s* can be converted into an estimated percentage of learning events that occurred by social transmission, %ST = 18.5–100%. There was no support for the influence of sex, social rank or age on the rate of social or asocial learning (all <30%; Supplementary Information section 4f and Supplementary Table 2).

When we broke the NBDA down into a two-stage learning process (first, learning to interact with the apparatus; second, learning to actually solve it), there was evidence that the rate at which chimpanzees started interacting with the task was related to the number of successes observed, and evidence that this effect was stronger in Group 2 (*s*_1_: model-averaged estimate, 0.40; 95% CI, 0.032–1.78; %ST, 5.3–30.6; *s*_2_ model-averaged estimate, 2.12; 95% CI, 0.742–9.09; %ST, 13.0–43.2; 95% CI for *s*_2_/*s*_1_, 1.4–85.3; Fig. 3). There was also evidence that the rate at which chimpanzees solved the task once they had started interacting with it was related to the number of successes observed: the *s* parameter had a model-averaged estimate of 0.023, with a 95% CI of 0.009–0.58 conditional on the top model, corresponding to a %ST of 8.1–47.4. For more details, see Supplementary Information section 4g (Supplementary Tables 3 and 4).

## Discussion

A sequential task was presented to two groups of chimpanzees in their natural (woodland) enclosures for prolonged periods (three months) to test whether the required know-how to solve the task could be innovated in the absence of social demonstrations. Upon observing that no chimpanzee succeeded in solving the task despite numerous attempts, we concluded that the skill was too difficult for chimpanzees to acquire without observing the relevant know-how and continued with testing whether the skill could be learned and transmitted through the groups by seeding one proficient conspecific model in each group. Crucially, this assessment would test whether chimpanzees are able to socially learn know-how that they fail to re-innovate themselves, which has been posited as a crucial capacity underlying CCE in humans^14,37^. One chimpanzee in each group was thus independently trained to function as a conspecific demonstrator for their group members during the subsequent social learning phase. We found that 14 of 66 naive chimpanzees learned the skill from the demonstrators within two months’ time and that social learning was acting on the chimpanzees’ skill-acquisition process. Each of the skill adopters observed successful task manipulations at least nine times prior to learning, and most of them observed many more of these manipulations. Furthermore, the more times chimpanzees observed the task being solved, the faster they were to start interacting with it, and the faster they were to solve the task once they had started interacting with it. In conjunction with the fact that the same chimpanzees did not solve the task when no relevant social information was available despite numerous attempts (the baseline phase), we conclude that the chimpanzees used social learning to acquire a skill that seems beyond their ‘zone of latent solutions’ (ZLS) and thus that chimpanzees can master copying-dependent forms^14^. This suggests that chimpanzees use know-how copying to expand their skill set, which has been argued to form an important mechanism underlying CCE^3,37,46^.

A recently published study by Koops and colleagues showed that wild chimpanzees who are not acquainted with nut-cracking techniques did not learn the skill when exposed to all necessary elements (that is, uncracked nuts, cracked nuts and cracking tools) for prolonged periods^38^. The authors took these findings as evidence for nut cracking being a socially learned tradition that individual chimpanzees cannot independently innovate. In other words, the tradition was concluded to be socially learned and transmitted over generations to become a mastery beyond the chimpanzees’ ZLS^38^. This study was subsequently criticized for failing to test the chimpanzees’ capacities to invent nut cracking, given that many naive chimpanzees may not have been motivated to crack the distributed nuts or even eat the ones that were already opened^39,40^. Moreover, the conclusion of the study was contested, because the findings did not provide direct evidence that the chimpanzees could socially learn the necessary skill to crack the nuts (for studies reporting on the diverse means by which chimpanzees can learn nut cracking, see refs. ^18,51,52^). The current study put this question to the test and found evidence that chimpanzees can copy a sequence of behaviours that seemingly falls outside their ZLS. The required skill did not readily transpire out of their imagined space of know-how^14^, despite ample opportunity to solve the task, a motivation to do so and a conducive ecological environment. We qualify the environment as conducive given that the chimpanzees were tested in a group setting (allowing for a natural dynamic of social influences, such as local and social enhancement) and in their forested home-range territory (in contrast to most tests on CCE in chimpanzees, which typically take place in constrained zoo settings; for example, see refs. ^53,54^). Yet, these conditions did not lead to a successful execution of the required task sequence, until it was seeded in the group by one trained conspecific model. Taken together with the NBDA results, this suggests that once the necessary information was available in their group, the chimpanzees used social learning to overcome the dead end of their individual attempts during the baseline phase. Whether the chimpanzees used know-how copying or social learning mechanisms with lower-fidelity transmission—for example, copying know-what and know-where—needs to be pinpointed by follow-up studies^31^ (also see ref. ^55^).

In this light, we note that both the transition from being task-naive to engaging with the apparatus and the transition from engaging to successfully executing the sequence were evidentially facilitated by social learning. This indicates that the chimpanzees not only were drawn to the apparatus by others’ actions (which probably played a role during the baseline phase as well) but also learned know-how from observing successful models. Despite the fact that our study design did not incorporate a two-action methodology^56^, the NBDA identified social learning as the most probable mechanism by which the chimpanzees acquired the necessary skill to operate the apparatus. Similar results were recently obtained when Barbary macaques were presented with a novel foraging task, although contrary to the current study, the required behaviour for these primates (that is, pushing a rotating door aside) was plausibly assumed to be within their behavioural repertoires, which led the authors to infer that most likely ‘response facilitation’ (that is, using a familiar action in a novel context) had been at play^57^. Experimental evidence regarding CCE in chimpanzees specifically remains contested—predominantly by ZLS proponents^22,26^—because, whereas social learning may be implicated in their skill adoption and/or improvement (for example, see refs. ^46,58,59^), these behaviours typically also get invented in control conditions without demonstrators (that is, the baseline in the current study). This means that those putative cumulative products do not really qualify as accumulation by collective action, which is one of the cornerstones of CCE in humans. The strength of our study lies in the fact that the chimpanzees did not invent the behaviour during the relatively long baseline period, and that the chimpanzees needed to learn an opaque sequence of behaviours rather than one behavioural action, which makes it substantially less probable that they would learn the solution to the task in the absence of relevant social information. Overarchingly, if the capacity to copy know-how becomes increasingly evidenced in chimpanzees, the ZLS argument that wild chimpanzee cultures should be regarded as collections of individual re-innovations based on parsimony^14^ should be re-evaluated. Moreover, it would mean that chimpanzees’ potential to develop cumulatively evolving cultures might not be hindered by a lack of know-how copying capacity, although it may be that chimpanzees rely on social information less markedly than humans do^60,61^.

The inference that the respective sequence of behaviours is beyond the chimpanzees’ ZLS rests on (1) the finding that despite ample time and attempts, none of the chimpanzees (*n* = 66) solved the task, and (2) the fact that a sequence of actions was required to solve the task, which was opaque and as such not likely to be invented by predispositions or individual learning attempts. Moreover, these actions needed to be executed in a specific temporal order, which substantially complicates the task. We acknowledge that although the chimpanzees may not have a genetic predisposition to solve the entire task, some of its required components may be (partly) predisposed. To avoid the possible influence of genetic predispositions (which would make the inference of social learning more difficult), future studies may use even more unnatural techniques to test the know-how copying hypothesis with the aim to explore the boundaries of great apes’ cognitive capacities^62^, all the while contextualized within their respective ecologies^63^. Nonetheless, the fact that no chimpanzee solved the task during a substantially large time window during the baseline phase (which is more than twice as long as our subsequent social learning phase, and thus, sensu the ZLS account, sufficiently long^31^), and the statistical signature of social instead of individual learning during the social learning phase, bolster our conclusion that the chimpanzees in the current study used social learning to master a skill that they could not readily invent by themselves and thus may be beyond their ZLS^14,31^.

Regarding the ZLS’s emphasis on know-how copying for understanding cultural evolution, we note that the interplay between know-how copying and CCE requires nuance. CCE comprises complex sequences involving individual innovations, moderating potentials for cue longevity^35,64^, social transmission and contingent reiterations of this intricate dynamic^1^. As there are many forms of social transmission by which the next (cultural) generations can obtain information to build on^6,65–67^, it is unlikely that social transmission an sich is the bottleneck hampering CCE in non-human animal species^3,37^. Specifically, know-how copying may not be the only mechanism by which CCE ensues (let alone the only prerequisite). Whereas know-how copying may be essential for certain aspects of culture (such as social norms or conventions), for other cultural manifestations (such as the usage of cultural artefacts), mechanisms yielding less-high-fidelity transmission may suffice^66,67^.

Furthermore, we note that culture is much more abundant within the ZLS, even in the human species. Long lists of traditional ways of behaving are well within each human’s capacity to innovate on their own—we just do not do so because we are culturally wired^2,4^. In other words, we absorb cultural information like sponges, even for behaviours we can easily execute on our own, making humans an intrinsically cultural species. Here, two aspects are relevant to point out: (1) these within-ZLS transmission events may bestow substantial fitness benefits, and (2) non-human animal species similarly possess within-ZLS culture. Regarding point 1, irrespective of know-how copying taking place, the transmission of innovations via social learning reaps benefits—either instrumental^4,68^ or social^33,69^—compared with a hypothetical control condition in which the behaviour may (or may not) be re-innovated by each and every individual alone, if only by the latency at which the implicated benefits become available. For instance, for chimpanzees, the nutritional value of nut cracking^70,71^, including its percolating effects on bodily fitness, survival and reproduction, would be missed out on for substantially longer in the absence of the capacity for social learning. Regarding point 2, the list of cultural traditions in, for example, chimpanzees^8,12,72^, bonobos^73–75^ and orangutans^76^ is steadily growing with increasing research efforts. Taken together, this means that the selective forces driving the emergence of know-how copying may not be the same as the ones driving the evolution of CCE (for a new proposal on the link between know-how copying and the emergence of CCE, see ref. ^17^). The value of focusing on know-how copying lies in its potential for catapulting CCE owing to its quality of retaining useful information in the population, but the evolution of CCE, from the adaptationist perspective, may depend on a broader range of capacities and processes^1,22,35^.

In conclusion, our study shows that chimpanzees can acquire a complex skill via social learning that had remained unattainable via asocial learning for an extended period, despite frequent attempts to obtain the associated food reward. After we introduced the behavioural solution into the groups via trained conspecific models, a strong signature of social learning in naive chimpanzees’ tendencies to attempt and obtain the complex skill was detected with advanced statistical techniques. We conclude that chimpanzees are capable of using social learning to acquire know-how that they cannot easily innovate by themselves.

## Methods

Our research complied with international standards (the Weatherall report), institutional guidelines (Chimfunshi Research Advisory Board: ref. 2016C027) and national standards for the treatment of animals as stipulated by the Zambia Wildlife Authority.

### Subjects

The subjects were 66 chimpanzees (43 females) housed in two separate groups at the Chimfunshi Wildlife Orphanage Trust, Zambia (Supplementary Table 5). Of the 66 residents, 61 chimpanzees (40 females) participated during the social learning phase (at least once as observer or active apparatus operator; for the details, see Table 1). At Chimfunshi, the chimpanzees live in large, forested (Miombo) enclosures^44^, stay outside overnight and only come indoors for supplemental feeding between 11:30 and 13:30 (for more details, see ref. ^77^, for example).

### Procedure

At time point *t*_0_ (in days), we placed an automated apparatus in the chimpanzees’ enclosures (one in each group) to be explored and operated by the chimpanzees. The apparatus consisted of a large plastic box with a drawer that could be pulled out to make a hole in the drawer accessible, which in turn needed to be filled with a wooden ball to trigger the mechanism that provided a food reward (Supplementary Video 1). The drawer was secured in the apparatus with a spring mechanism, which caused the pulling to be effortful, whereas the pushing back into the apparatus occurred mechanically and almost automatically. The food reward consisted of a few peeled peanuts (five to eight units at a time). To enable ample opportunity for all chimpanzees to find and use the wooden balls, we provided the balls in large quantities (~75 balls) distributed over a circular area (radius ~25 metres) with the implemented apparatus in its centre. Most balls were distributed close to the apparatus such that the chimpanzees had them readily available when they were exploring the apparatus (Fig. 1c). The apparatus was equipped with a motion-sensitive GoPro camera, which recorded all events immediately surrounding and interacting with the apparatus. At regular occasions (about once a week), the chimpanzee caretakers entered the enclosure to check whether the mechanisms were all still working and to check whether any balls had been successfully inserted into the apparatus, in which case our crucial baseline (no chimpanzee will be able to operate the apparatus successfully by themselves—that is, without any social demonstrations) would have failed.

The apparatuses including the wooden balls were made accessible to the chimpanzees for 3 months in each group, which thus amounts to approximately 3 months × 30 days × 22 hours per day × 2 groups = 3,960 hours of baseline testing for 66 chimpanzees (we count 22 hours instead of 24 hours because most chimpanzees spend ~2 hours indoors each day). During this period, the GoPro cameras recorded the attempts to solve the apparatus. Across the entire baseline period, not a single chimpanzee was successful in operating the apparatus through the drawer mechanism once.

Subsequently, the apparatuses remained in the chimpanzees’ enclosures for another eight months, during which the apparatus was irregularly checked (that is, peanuts, balls and working mechanism) by the local caretakers at Chimfunshi. During this period, the wooden balls remained in the enclosure, and the apparatuses were fully functional. Yet, similar to the controlled baseline, not a single chimpanzee solved the task even once, which we inferred from the fact that not a single wooden ball was ever found inside the apparatus (where it would have ended up if the task had been executed successfully). Given that this eight-month time window was not directly monitored by the experimenters, however, we conservatively refrained from considering this second baseline phase in our analyses.

At *t*_331_, we selected one chimpanzee per group to function as a demonstrator for the other chimpanzees in the social learning phase. In each group, we chose an adult female of middle/high rank and kept them inside their indoor holding facility after their daily supplemental feeding (between 11:30 and 13:30) for a training session of ~20 minutes, and we repeated this sequence for eight consecutive days (*t*_331–338_). During each training session, outside the visual fields of the rest of the group, we let the chimpanzee explore the apparatus freely for some minutes, after which we provided cues about the workings of the apparatus—for example, by pushing out the drawer from the human side of the room (through the mesh) and by operating the drawer with the wooden balls. Both the selected chimpanzees did not immediately learn the contingencies of the apparatus (which further attests to the empirical fact that the skill to operate the apparatus was difficult for the chimpanzees to acquire), but they were successful and motivated to use the apparatus after the eight training sessions. We chose chimpanzees of middle/high rank because these chimpanzees would be able/allowed by the group to operate the apparatus with others around. We acknowledge that specific individuals as demonstrators may affect social transmission differently, but note that this possibility is irrelevant here given that our research question pertained to whether or not social transmission would ensue an sich.

At *t*_339_, we made the apparatus available to the entire group, including the trained demonstrators, by attaching it to the mesh connecting the chimpanzees’ indoor facility to their forested outdoor enclosure. To ensure proper functioning of the apparatus and allow for fine-grained coding of observations and attempts (including successes) by the chimpanzees to operate the apparatus, an experimenter provided the rewards into the designated compartment upon a chimpanzee successfully inserting a wooden ball into the drawer. To allow the chimpanzees the possibility to operate the apparatus, wooden balls were thrown into the chimpanzees’ enclosure, both in the vicinity of the apparatus and further away in the forest (for the number of wooden balls made available to the chimpanzees across experimental time, see the Supplementary Information). Besides serving as an essential part in the behavioural sequence to be learned, the idea behind using wooden balls was that the chimpanzees needed to leave the apparatus after successful insertion if they wanted to re-use the apparatus. This requirement provided ample space and time for other chimpanzees (for example, shy or low-ranking individuals) to investigate and attempt the apparatus. Across a period of two months, in each group, we ran 39 two-hour test sessions.

### Coding

A successful operation of the apparatus was coded when a chimpanzee managed to insert a ball into the drawer and the drawer was reinstated into the apparatus, which caused the ball to (audibly) fall into the apparatus. Attempts to operate the apparatus were coded when chimpanzees were interacting with the apparatus (for example, fiddling with the drawer or bouncing balls against the apparatus) but not to the point of succeeding. Observations were coded when an individual resided within 1.5 metres of the chimpanzee successfully operating the apparatus when the solution was executed. Given that the successful operations occurred in a designated space in front of the apparatus—in the eye-level meshwork window of the chimpanzees’ outdoor enclosures—it was straightforward to code observations. A second coder naive to the project coded 20% of the data from video—specifically, who successfully operated the apparatus and who was observing at that time. The coders agreed 91.9% of the time on the identity of solvers and 85.0% of the time on the identity of observers.

### Statistical analysis

The data were analysed using NBDA in the R statistical environment v.3.6.1 (ref. ^47^) using the NBDA package v.0.8.3 (ref. ^48^). NBDA infers social transmission if the diffusion follows the connections of a social network representing opportunities to learn between members of each dyad^49,50^. We used the order-of-acquisition variant of NBDA^50^, which takes as data only the order in which individuals acquire the target behaviour and not the times of acquisition. The full specifications of all models and additional explanations are given in the Supplementary Information.

To assess whether social transmission was operating and to elucidate the conditions under which it occurred, we considered models with two social networks representing the changing pattern of observations over time. The ‘absolute observation’ network gave the number of times individual *i* had previously observed *j* successfully solving the task^78^ and represents the hypothesis that the rate of social transmission is proportional to the number of times *i* has observed the task being solved^79^. The ‘individuals observed’ network (1, *i* has observed *j* solve the task; 0, otherwise) assumed instead that the rate of social transmission was determined by the number of individuals *i* had observed solving the task, regardless of how many times each individual was observed. We also considered models in which the social network was replaced with a binary variable indicating whether or not *i* had seen at least one other chimpanzee solve the task prior to time *t*, thus representing the hypothesis that a single observation was sufficient for social transmission to occur. Finally, we ran models containing a static group network indicating which individuals were in the same group (1) or different groups (0). This was to test whether a positive result for the networks above indicated evidence that the diffusion followed the pattern of observations within each group above as opposed to simply being a result of group differences in relative time of acquisition^6^. These four networks were entered into the model separately, and their fit to the data was compared.

Sex (0, female; 1, male), age and social rank were included as individual-level variables (ILVs) that potentially influence the task solving order. Rank was determined by averaging the ordinal rank assessments as independently provided by three experienced (at least eight years) caretakers at Chimfunshi. We used the ‘unconstrained’ model to include the effects of ILVs; this model independently estimates the effects each ILV has on asocial and social learning^6^. We used a multi-model inference approach using Akaike’s information criterion corrected for sample size^80^ to obtain support for models using the absolute observation network, the individuals observed network, the single observation network and the group network. For each observation network, we fitted models representing the following hypotheses: (1) social transmission of different strength in each group, *s*_1_ ≠ *s*_2_; (2) social transmission of equal strength in each group, *s*_1_ = *s*_2_; (3) social transmission only in Group 1, *s*_2_ = 0; and (4) social transmission only in Group 2, *s*_1_ = 0. For the group network, only models representing hypothesis 2 were fitted, since this was only intended as a null hypothesis for comparison with other combinations of networks and hypotheses 1–4. For each observation network and for hypotheses 1–4, we fitted models with every combination of the three ILVs affecting asocial and social learning, resulting in 16 models for each set. For the asocial-learning-only set (hypothesis 5), *γ* parameters have no effect and so were excluded, resulting in only four models. We calculated the total Akaike weight as a measure of support for each hypothesis 1–4 and each network^80^. Due to the lower number of models in the asocial set (hypothesis 5), we did not use the total Akaike weight as a measure of support for asocial learning; instead, we used the 95% CIs for the *s* parameters to this end.

To further elucidate the role of social learning in the diffusion, we used the multistate extension of NBDA^81^, in which chimpanzees moved from a naive state (have never manipulated the task) to an interacting state (have started manipulating the task but not yet solved it) to an informed state (have solved the task at least once), using the absolute observation network favoured in the simple NBDA. If individuals were initially attracted to the task by observation of other individuals solving it, we would expect a social effect on the transition from naive to interacting. If the rate at which chimpanzees transition from interacting to informed is related to the number of times they have observed successful interactions with the task, it suggests they may be learning something about how to solve the task. Potentially, both processes could operate in tandem (see the Supplementary Information for the model specifications).

### Analysis of observation network structure

The NBDA estimated the social learning effect per observation and assessed whether this differed between the two groups. Another way in which the groups might differ is in the pattern of who observes whom. We tested for patterns in the observation network—specifically, (1) whether chimpanzees were more likely to watch maternal kin solve the task than non-kin individuals, (2) whether there was a bias towards observing older/younger individuals and (3) whether there was a bias towards observing higher/lower ranks. We fitted a generalized linear mixed model with a logit link function in which each potential observation between a manipulator and a potential observer was included as binary data point (1, observation; 0, no observation) with random effects accounting for variation among observers and manipulators in their propensity to observe and be observed. We also allowed for the possibility that a manipulation was more likely to be observed by *i*, if *i* had observed the previous manipulation. We used Bayesian inference, using Markov chain Monte Carlo methods to obtain a posterior sample for the model parameters^82^. This was done using JAGS^83^ run via the R statistical environment using the runjags package v.2.0.4 (ref. ^84^) (see the Supplementary Information for the full details). We estimated odds ratios for each effect in each group as the back-transformed mean of the posterior distribution, as well as the ratio of effects in each group, both with 95% highest posterior density intervals.

### Ethics

Animal husbandry and research protocols complied with international standards (the Weatherall report), institutional guidelines (Chimfunshi Research Advisory Board: ref. 2016C027) and national standards for the treatment of animals as stipulated by the Zambia Wildlife Authority. The Chimfunshi Research Advisory Board reviews projects for chimpanzee safety and welfare, and it functions as an independent entity for evaluating ethical and feasibility criteria for each study proposed to be conducted at Chimfunshi since 2011.

### Reporting summary

Further information on research design is available in the Nature Portfolio Reporting Summary linked to this article.

### Supplementary information

Supplementary Information Supplementary Fig. 1, Tables 1–5 and Methods.

Reporting Summary

Supplementary Video 1 Demonstration of the required skill.

Supplementary Video 2 Chimpanzees attempting the skill.

Supplementary Video 3 Chimpanzees attempting the skill.

Supplementary Video 4 Chimpanzees attempting the skill.

Supplementary Video 5 Chimpanzees attempting the skill.

Supplementary Video 6 Chimpanzees attempting the skill.

## Data Availability

All data used in this study are available at a public repository: https://datadryad.org/stash/share/FF8SI1kkbLQaVFvsG4BR6bXdJoxGfYTabBkygxx1pvM.

## Code availability

The code used in this study is available upon request.
