# Supplementary material for: Chimpanzees use social information to acquire a skill they fail to innovate
Source: Nat Hum Behav. 2024 Mar 6;8(5):891–902. doi: 10.1038/s41562-024-01836-5 (PMC11132989; doi:10.1038/s41562-024-01836-5)
Supplement: Supplementary file 1 — Supplementary Fig. 1, Tables 1–5 and Methods. [file 41562_2024_1836_MOESM1_ESM.pdf]

nature human behaviour

Article

https://doi.org/10.1038/s41562-024-01836-5

Chimpanzees use social information to acquire a skill they fail to innovate

In the format provided by the authors and unedited

Table of contents

Included in this PDF:

Methods

1. *Distribution of wooden balls*
2. *Rewarding*
3. *Training of the model*
4. *Statistical analysis (NBDA)*
   a. *Formal model specification*
   b. *Observation networks*
   c. *Inference*
   d. *Comparison of networks*
   e. *Model-averaged estimates of effects of ILVs*
   f. *Two-stage learning process*
      i. *Formal model specification*
      ii. *Inference*
      iii. *Naïve -> interacting results*
      iv. *Interacting -> solved results*

Figures and Tables

Methods

1. Distribution of wooden balls

The chimpanzees required wooden balls to operate the apparatus successfully; hence, we provided the balls to them. First, throughout the baseline phase (*Part 1*), the balls were plentiful available in the chimpanzees' enclosure (±75 at all times), distributed over a concentric area with a radius of ± 25 meters with the implemented apparatus in its centre-point. Most balls were distributed close to the apparatus such that the chimpanzees had them readily available when they were exploring the apparatus (Figure 1c in *Main text*). The chimpanzees were rather naïve to such experimental testing (except for a study on prosocial fruit-juice provisioning[1]), yet they exhibited curiosity for the apparatus and likely gained motivation to operate the apparatus because they could both see and smell the food rewards inside the box (peanuts). Moreover, in each group, the chimpanzees experienced the presence of the food inside the box in their first week of the baseline phase (see *Main text*).

During the experimental sessions (*Part 2*), we used the exact same apparatus as during the baseline phase, but instead of leaving the apparatus full-time in the enclosure, for the experiment, we attached the apparatus to the mesh. We did this because *i*) for the training of the models, we had to get the apparatus out of the enclosure and put it in the indoor holding facility to facilitate the training (without others seeing it), and *ii*) we anticipated that the chimpanzees would become proficient now that a model had been trained, which meant that both the wooden balls and the food had to be replenished regularly (while we cannot go inside the chimpanzees' outdoor enclosures regularly). Moreover, we wanted to be sure to obtain a full record of what the chimpanzees had observed before attempting or succeeding themselves, which would not have been possible outside, because of the limited range of the Go-Pro images and the fact that the apparatus could be solved 24/7, which would have been a further challenge for the Go-Pros (no night vision and limited battery).

Indeed, during *Part 2*, we found that the chimpanzees were functionally using the wooden balls (first only the trained models, but afterwards also the formerly naïve chimpanzees, see Figure S1, and Figures 2 and 3 in the *Main text*), thus the balls were provided by the experimenters in a continuous effort. The experimenters adhered to the following ball-replenishing procedure: the first two sessions, mainly the trained model was directly provisioned (i.e., by throwing) to entice them to act as demonstrators for the rest of the group (Figure S1). In the following sessions, we provided Group 1 with ±40 balls and

Group 2 with ±80 balls at all times, meaning that whenever a ball was used to operate the apparatus, this ball was readily thrown back into the enclosure (this later transitioned into piles of ±5-10 balls, before throwing them back in). Importantly, at all times, we aimed to provide all group members with at least one ball as to give all the chimpanzees the opportunity to solve the apparatus and thus show us that they had acquired the skill at stake. We also note that the experimental sessions lasted for only 2 months not because the number of individuals learning the skill reached a plateau, but because of logistical constraints.

2. Rewarding

Upon successfully entering the wooden ball into the cavity in the pulled-out drawer, the chimpanzee was rewarded. At first, we rewarded with small amounts of peanuts (sessions 1-5), but because of ensuing aggression over the peanuts, we decreased this by rewarding only one peanut (sessions 6-15) per solve, after which we transitioned to rewarding one corn-puff (i.e., a ±2cm large somewhat salted crisp) per solve (sessions 16-39). We provided these corn-puffs manually given that the automated mechanism could not take squishy food items. The puffs were put in the rewarding slide by a human experimenter. Given that the rewarding (distribution) itself was not under experimental scrutiny, this procedure worked adequately. In other words, even though the adjustments in the rewarding scheme may have altered some chimpanzees' motivation to partake in the experiment, the fact that chimpanzees learned the skill at all remains unchanged. Moreover, we started off during the experimental phase with shelled peanuts (same as in baseline), which the chimpanzees like to eat very much. In fact, they liked the peanuts so much that they started fighting over them, which was the reason for us to switch to a bigger, one-unit reward (corn-puff). If anything, the corn-puffs may not have been to the liking of all chimpanzees, or at least less so than the preferred peanuts, which would have biased our results towards less chimpanzees being motivated to learn the skill.

3. Training of the model

The model of each group (Rita in Group 1; Pippa in Group 2) was trained on the apparatus in isolation from the group to ensure that no other group member could benefit from the human instructions. Both chimpanzees took approximately 8 sessions (of ±20 minutes each) to fully understand the contingencies of the apparatus, which we operationalized as at least 8 successful operations on the apparatus out of 10 successive opportunities (i.e., provisioning of a wooden ball). The training of the models comprised various techniques including enhancing the relevant parts of the apparatus (e.g., the drawer), gesturing towards the wooden

balls, and demonstrating the entire sequence to the chimpanzees (this was done by the resident caretakers). The selection of the models was based on our assessment of them being able/willing to operate the apparatus in the presence of the entire group. Both females were of mid/high rank and endowed with sufficient confidence to endure the group's pressure.

4. Statistical analysis (NBDA)

Network-based diffusion analysis (NBDA) infers social transmission of novel behaviour if the pattern of its spread follows a social network, which is taken to represent opportunities to learn from others[2]. Here, we used a dynamic observation network such that the connection from *i* to *j* in group *k* at time *t* ($o_{k,ij}$) was the number of times *i* had observed *j* successfully solving the task prior to time *t* (see[3]), since this type of network provides the most direct quantification of opportunities to learn from others by observation[4]. We also used other networks derived from the direct observation network (see below).

We used the order of acquisition (OADA) variant of NBDA[5], which takes as data only the order in which individuals acquire the target behaviour and not the times of acquisition. This has the advantage that it does not make any assumptions about the baseline rate function (see Eqn. S1 below) unlike the time of acquisition variant (TADA). TADA can have more statistical power when the assumed baseline hazard function is correct, but can have lower power or inflated false positive rate when it is incorrect[5].

Since we have data on the diffusion of each behaviour through more than one group, one option is to treat these as separate diffusions with separate baseline rate functions. However, we chose to include all groups in the same stratum, with the same baseline rate function- i.e., to treat them as one diffusion, but with zero connections between individuals in different groups to indicate their lack of opportunities to learn from one another[6]. This approach has the advantage that it is sensitive to the behaviour diffusing through different groups at different times: if the behaviour spreads through one group, then another group, etc.; this is consistent with social transmission of the behaviour. In some cases, this pattern might also be consistent with groups differing in their rate of asocial learning of the target behaviour, making it necessary to fit different rates of asocial learning to each group to control for this possibility. However, given the nature of the target behaviours in this case, we can *a priori* rule out the possibility that groups substantially differ in asocial learning rate, especially given that no chimpanzee in either group solved the apparatus without social

information (i.e., during the *baseline* phase). To be sure, we also ran a normal OADA, which is sensitive only to the order within each diffusion. In a normal OADA, a spurious result cannot arise as a result of group differences in asocial learning rate. Here, we obtained the same results: evidence for social transmission (and no evidence for a difference between the groups), which adds to our confidence that we have evidenced a true social transmission effect.

Our primary goal with the NBDA was to establish whether there was evidence for social transmission of the task solution, determined by the extent to which the diffusion followed the pattern of observations over time. We furthermore assessed whether three individual-level variables (ILVs), namely age, sex and rank, affected the rate of asocial or social learning.

a) Formal model specification

The full model be expressed as follows:

$$\lambda_i(t) = \lambda_0(t)\left[\left(s_1 \sum_j o_{1,ij}(t) + s_2 \sum_j o_{2,ij}(t)\right) exp\left(\gamma_1 x_{1,i} + \gamma_2 x_{2,i} + \gamma_3 x_{3,i}\right) + exp\left(\beta_1 x_{1,i} + \beta_2 x_{2,i} + \beta_3 x_{3,i}\right)\right](1 - z_i(t))$$

Eqn. S1

Where $\lambda_i(t)$ is the rate at which individual *i* first solves the task at time *t*, $\lambda_0(t)$ is the unspecified baseline rate function, $o_{k,ij}(t)$ is the number of times *i* has observed *j* in group *k* prior to time *t* (set to 0 when *i* or *j* are not in group *k*), s_k is a fitted parameter estimating the relative rate of social transmission per observation in group *k*, $x_{1,i}$ is the sex of *i* (0= female, 1= male), $x_{2,i}$ is the standardized age of *i*, $x_{3,i}$ is the transformed rank of *i* (see below); β_m are fitted parameters estimating the effect each ILV has on asocial learning, whereas γ_m estimate the effect on social learning. Rank (x_3) was transformed such that 0= highest ranked in each group, 1= lowest rank within each group with other ranks equally spaced within each group, meaning β_3 and γ_3 estimate the difference between highest and lowest ranking chimpanzees.

Given that we tested two groups, we had several options regarding assumptions on social transmission rates. The variant of no difference in social transmission rate, per observation,

between groups 1 and 2 is represented by models with the constraint $s_1 = s_2$, and the hypothesis of no social learning in each group can be represented by $s_1 = 0$ and $s_2 = 0$.

b) Observation networks

We wished to investigate the conditions under which social transmission occurred. One hypothesis is that social transmission to *i* occurs at a rate proportional to the number of observations, represented by the ***absolute observation network*** described above. Here, $\sum_j o_{1,ij}(t)$ reduces down to the number of times *i* has observed the task being solved prior to time *t*. Alternatively, chimpanzees might learn socially at a rate proportional to the number of individuals they have observed solving the task, regardless of the number of times they have observed each individual. This hypothesis was represented by the ***individuals observed network*** in which $o_{k,ij}(t) = 1$ if *i* has observed *j* solve the task at least once prior to time *t*, such that $\sum_j o_{1,ij}(t)$ is now the number of individuals *i* has observed solving the task prior to time *t*. Next, we reasoned that a single observation of any chimpanzee solving the task might be sufficient for social transmission to occur, with any further observations making no difference. This hypothesis was represented by a model in which $\sum_j o_{1,ij}(t)$ was replaced with a binary indicator variable, $o_{1,i}(t)$, taking the value 1 if *i* had observed at least one chimpanzee solving the task prior to time *t* and 0 otherwise (***single observation network***). Finally, we ran models containing a static ***group network*** indicating which individuals were in the same (1) or different groups (0). This was to test whether a positive result for the networks above indicated evidence that the diffusion followed the pattern of observations within each group above as opposed to simply being a result of group differences in relative time of acquisition[6]. These four networks were entered into the model separately (one for each group, *k*) and their fit to the data was compared (see section c below).

c) Inference

We used a multi-model inference approach using Akaike's Information Criterion corrected for sample size (AIC_c)[7] to obtain support for models using the absolute observation network, individuals observed network, single observation network and group network. For each observation network, we fitted models representing the hypotheses: a) social transmission of different strength in each group, $s_1 \neq s_2$ b) social transmission of equal strength in each

group, $s_1 = s_2$; c) social transmission only in group 1, $s_2 = 0$; and d) social transmission only in group 2, $s_1 = 0$. For the group network, only models representing b) were fitted, since this was only intended as a null hypothesis for comparison with other combinations of networks and a-d.

For each observation network and for a-d we fitted models with every combination of 3 ILVs affecting asocial and social learning, resulting in 16 models for each set. For (e) asocial learning, γ parameters have no effect so were excluded resulting in only 4 models. We calculated the total Akaike weight as a measure of support for each hypothesis a-d and each network[7]. Due to the lower number of models in the asocial set (e) we do not use the total Akaike weight as a measure of support for asocial learning, instead we use the 95% confidence intervals for the *s* parameters to this end (see below).

For the favoured network, we calculated model averaged estimates, unconditional standard errors, and the total Akaike weight for the effect of each ILV on asocial learning (β parameters) and social learning (γ parameters). In some models, standard errors could not be derived. When calculating the unconditional standard error, the standard errors for these models were replaced with a Akaike-weighted mean across models with a standard error, allowing an approximate unconditional standard error to be calculated.

Standard errors are often a misleading measure of precision for parameters in an NBDA, since such parameters often have much higher precision for a plausible lower limit than for a plausible upper limit or vice versa. Consequently, we obtained 95% confidence intervals (CIs) for parameters with Akaike weight >50% using the profile likelihood method, using the model with best AIC_c. Since *s* parameters are difficult to interpret directly, we also obtained an estimate of the number of learning events that are predicted to have occurred by each pathway corresponding to the estimate for each *s* parameter and its 95% Confidence Interval[8].

d) Comparison of networks

Table S1 below shows the support for each hypothesis a-d and each network considered.

Table S1. The support (total Akaike weight) for each network and hypothesis combination. Each cell represents a set of 64 models except asocial learning (*) which has only 8 (see section c). Totals are provided for the three observation networks with equal numbers of models.

**Network**	Total	$s_1 \neq s_2$	$s_1 = s_2$	$s_1 > 0$ $s_2 = 0$	$s_1 = 0$ $s_2 > 0$	$s_1 = s_2 = 0$
Absolute observation	50.4	8.2	39.2	2.6	0.4	-
Individuals observed	34.7	5.0	27.7	1.8	0.2	-
Binary observation	13.5	1.5	9.5	2.3	0.2	-
Group network		-	0.6	-	-	-
Asocial learning		-	-	-	-	0.6*

The results support the hypothesis that the rate of social transmission was best predicted by the number of task solutions observed (support= 50.4%), as opposed to the number of individuals observed solving the task (support= 34.7%). They also suggest that a single observation of a task solution was not sufficient for a full social learning effect to occur (support= 13.5%). Models in which $s_1 = s_2$ were best supported suggesting that social transmission occurred in both groups, and that there was no evidence of a difference in the magnitude of the social effect between groups. The absolute observation network with $s_1 = s_2$ received 65.3x more support than the group network, further supporting the hypothesis of social transmission following the pattern of observations within each group. Asocial learning also receives relatively little support at 0.6%, however, only 4 models were fitted (c.f. 16) so we prefer to use the 95% C.I.s for *s* (see below) to quantify the strength of evidence against purely asocial learning ($s_1 = s_2 = 0$).

e) Estimates of social transmission effects

Estimates of social effects were made conditional on the absolute observation network since this network received the most support. For a dynamic observation network, the *s* parameter estimates the increase in rate of solving per observation, relative to the baseline rate of asocial learning (set to a female of mid rank and age). It was found that the log-likelihood levelled out to an asymptote as *s* tended to infinity, meaning we are unable to generate a single maximum likelihood estimate for $s = s_1 = s_2$ since a large range of values up to infinity explain the data equally well. However, conditional on the best fitting model, the 95% C.I. for *s* was 0.461 – Infinity, meaning the data provides a lower plausible limit on the size of *s*.

The 95% C.I. for s can be converted into an estimated percentage of learning events that occurred by social transmission, %ST= 18.5 – 100%. Since this range is conditional on the single best fitting model, we also calculated the %ST corresponding to the lower 95% confidence limit in all models with $s = s_1 = s_2$ to determine the robustness of the estimate to model selection uncertainty of ILVs. We calculated the Akaike weighted average for the lower limit of %ST at 15.7%. In all models except the lowest ranked model in the set (Akaike weight= 5e-7), the lower 95% limit for s was estimated at >0. So overall, from the basic NBDA described here, we have strong evidence that social transmission of the task solution occurred.

f) Model-averaged estimates of effects of ILVs

Table S2 below shows the effects of the individual-level variables (sex, age, and rank) on asocial and social learning.

Table S2. Model averaging for individual-level variables in the NBDA conditional on the absolute observation network.

	Model-averaged estimate	Unconditional SE*	Back-transformed effect[ł]	Total Akaike weight
**ILV effects on asocial learning**				
Sex (female-male)	0.00	0.00	1.00x	0.206
Age (per SD)	-2.83[$]	811[$]	0.06x	0.264
Rank (bottom-top)	-0.44	6.5e4[$]	0.64x	0.189
**ILV effects on social learning**				
Sex (female-male)	0.24	0.27	1.27x	0.278
Age (per SD)	-0.10	0.24	0.90x	0.236
Rank (bottom-top)	0.20	0.17	1.22x	0.172

*Unconditional standard errors (USE) are approximate, see section (c). [ł] Since effects of ILVs are estimated on the log scale, the back-transformed effects give the multiplicative effect of one unit change in the ILV. [$]The USE for age and rank are very high- this is due to one or more models of low weight with a flat log-likelihood resulting in a high SE, and thus a high USE overall. This has likely also skewed the estimate of the effect of age to an unrealistically high coefficient despite its low Akaike weight.

Since none of the effects of the ILVS had high support (all well under 50%), we did not derive 95% C.I. for any ILV effects using the profile likelihood method. Overall, there is little or no evidence that these variables affected social or asocial learning, though the standard errors also suggest that the data also cannot rule out a sizeable effect.

g) Two-stage learning process

In a standard NBDA, individuals are modelled as moving from a naïve state (never solved the task) to a solved state (solved the task). Here, we used the multistate extension of NBDA[9] in which chimpanzees moved from a **naïve** state (never manipulated the task) to an **interacting** state (have started manipulating the task but not yet solved it) to a **solved** state (have solved the task at least once). This involves breaking the analysis down into two models, one for modelling the rate of transition to **naïve->interacting** and another modelling the transition from **interacting->solved**. Breaking the analysis up in this way offers more insight into the role of social learning in the diffusion of behaviour. If individuals are initially attracted to the task by observation of other individuals solving it, we would expect a social effect on naïve->interacting. If the rate at which chimpanzees transition from interacting->solved is related to the number of times they have observed successful interactions with the task, it suggests they may be learning something about how to solve the task. Potentially, both processes could operate in tandem. In three-spined sticklebacks learning to solve a foraging task, evidence was only found of an effect on the former transition[9].

i. Formal model specification

The full model used for the naïve-> interacting (N->I) transition can be expressed as follows:

$$\lambda_{NI,i}(t) = \lambda_{NI,0}(t)\left[\left(s_{NI,1}\sum_j o_{1,ij}(t) + s_{NI,2}\sum_j o_{2,ij}(t)\right)exp\left(\gamma_1 x_{1,i} + \gamma_2 x_{2,i} + \gamma_3 x_{3,i}\right) + exp\left(\beta_1 x_{1,i} + \beta_2 x_{2,i} + \beta_3 x_{3,i}\right)\right](1 - I_i(t))$$

Eqn. S2

This is simply the same form as for the NBDA shown in Eqn. S1, but with the subscript NI added where appropriate to show that the rate of transition from naïve to interacting is being

modelled and $z_i(t)$ is replaced with $I_i(t)$, indicating whether *i* has interacted with the task at least once by time *t*. The data are modelled in the same way as for a standard NBDA, but instead of predicting the order in which individuals first solve the task, the model is fitted to predict the order in which individuals first interact with the task.

The full model used for the interacting-> solved (I->S) transition can be expressed as follows:

$$\lambda_{IS,i}(t) = \lambda_{IS,0}(t)\left[\left(s_{IS,1}\sum_{j} o_{1,ij}(t) + s_{IS,2}\sum_{j} o_{2,ij}(t)\right)exp(\gamma_1 x_{1,i} + \gamma_2 x_{2,i} + \gamma_3 x_{3,i}) + exp(\beta_1 x_{1,i} + \beta_2 x_{2,i} + \beta_3 x_{3,i})\right](1 - z_i(t))I_i(t)$$

Eqn. S3

This is the same form as for the NBDA shown in Eqn. S1, but with the subscript IS added where appropriate to show that the rate of transition from interacting to solved is being modelled and with the addition of $I_i(t)$. The addition of $I_i(t)$ ensures that only individuals that have entered the interacting state are ‘at risk’ of solving the task (in survival analysis terminology). Thus, as with a standard NBDA, we model the order with which individuals solve the task, but they effectively only enter the diffusion and become at risk of being the next individual to solve once they have started interacting with the task- this is equivalent to individuals entering the population at different times in a standard NBDA.

ii. Inference

We used multi-model inference to analyse each transition separately, as described in (c) above, except we only considered the absolute observation network. Our aim here was to break down the result found in the standard NBDA to offer more insight into the underlying social influences, so we limited our analysis to the network favoured in the standard NBDA.

For the NI model, there were a number of ‘true ties’: i.e., chimpanzees that started interacting with the task at the same time, or too close in time for the order to be resolved[5]. We fitted the full set of models accounting for true ties to generate the support for each hypothesis, however, this is highly computationally intensive since it requires calculating and adding likelihoods for all possible orders consistent with the tied data. Consequently, we refitted the

models ignoring the true ties and found they made a negligible difference to the results in this case, so we ignored true ties in order to calculate 95% C.I.s for each parameter.

iii. Naïve -> interacting results

Table S3. The support (total Akaike weight) for each social learning hypothesis for the naïve -> interacting transition. Each cell represents a set of 64 models except asocial learning (*) which has only 8 (see section c above).

**Network**	$s_1 \neq s_2$	$s_1 = s_2$	$s_1 > 0$ $s_2 = 0$	$s_1 = 0$ $s_2 > 0$	$s_1 = s_2 = 0$
Absolute observation	48.3	32.4	1.9	17.3	-
Asocial learning	-	-	-	-	0.0*

The greatest support was obtained for social transmission in both groups, but with a greater rate of transmission per observation in group 2. There was little support for an effect of any of the three ILVs on social or asocial learning (all <40%).

s_1 had a model-averaged estimate of 0.40, with a 95% C.I.= 0.032-1.78 conditional on the top model, corresponding to %ST = 5.3 – 30.6. Therefore, there is reasonable evidence of an effect of social transmission in group 1, however, this is not robust to model selection uncertainty: in 29/64 models the 95% C.I. for s_1 included zero. We conclude there is weak evidence of an effect of social transmission in group 1, but that this effect is unlikely to be highly important with most individuals starting to interact with the task asocially.

s_2 had a model-averaged estimate of 2.12, with a 95% C.I.= 0.742-9.09 conditional on the top model, corresponding to %ST = 13.0 – 43.2. In all 64 models the 95% C.I. for s_2 excluded zero, showing the finding is robust to model selection uncertainty. The model averaged 95% lower limit for %ST=16.8. Furthermore, the 95% C.I. for s_2/s_1 = 1.4 – 85.3, confirming the finding that social transmission was stronger per observation in group 2. We conclude there is strong evidence of an effect of social transmission in group 2, but that it is nonetheless likely that most chimpanzees started interacting with the task asocially.

Overall, it seems likely that that most chimpanzees would have eventually interacted with the task without having observed a trained demonstrator or another chimpanzee solve the task

first. However, there is strong evidence that this process was sped up by a social effect in group 2, with chimpanzees that observed successful manipulations interacting with the task sooner. Evidence for such an effect in group 1 was weaker, with evidence that, if it did exist, it was a weaker effect than in group 2.

iv. Interacting -> solved results

Table S4. The support (total Akaike weight) for each social learning hypothesis for the interacting-> solved transition. Each cell represents a set of 64 models except asocial learning (*) which has only 8 (see section c above).

**Network**	$s_1 \neq s_2$	$s_1 = s_2$	$s_1 > 0$ $s_2 = 0$	$s_1 = 0$ $s_2 > 0$	$s_1 = s_2 = 0$
Absolute observation	8.6	44.6	4.0	30.7	-
Asocial learning	-	-	-	-	12.0*

The greatest support was obtained for equal social transmission in both groups (44.6%), however, models with social learning only in group 2 (30.7%) also received comparable support. Consequently, we make inferences about $s = s_1 = s_2$, but also derive 95% C.I. for s_2/s_1.

s had a model-averaged estimate of 0.023, with 95% C.I.= 0.009-0.58 conditional on the top model, corresponding to %ST = 8.1 – 47.4. Therefore, there is reasonable evidence of an effect of social transmission. The finding is fairly robust to model selection uncertainty: in the 11 top models, accounting for 71% of total Akaike weight, the 95% C.I. for s does not include zero, but in 23/62 lower-ranked models the 95% C.I. includes zero. The model averaged 95% lower limit for %ST= 4.4. We conclude there is reasonable evidence of an effect of social transmission across both groups.

In the top model with $s_1 \neq s_2$, s_2/s_1 was estimated at 7.9 and the lower bound for the 95% C.I was 0.862. The upper bound could not be found since numerical errors were triggered when calculating the profile likelihood for $log(s_2/s_1)$> 4.5, however, we know that exp(4.5) = 90 is within the 95% C.I. for s_2/s_1, so we can rule out all but a small difference in favour of s_1, but cannot rule out a large difference in favour of s_2.

Figures and Tables

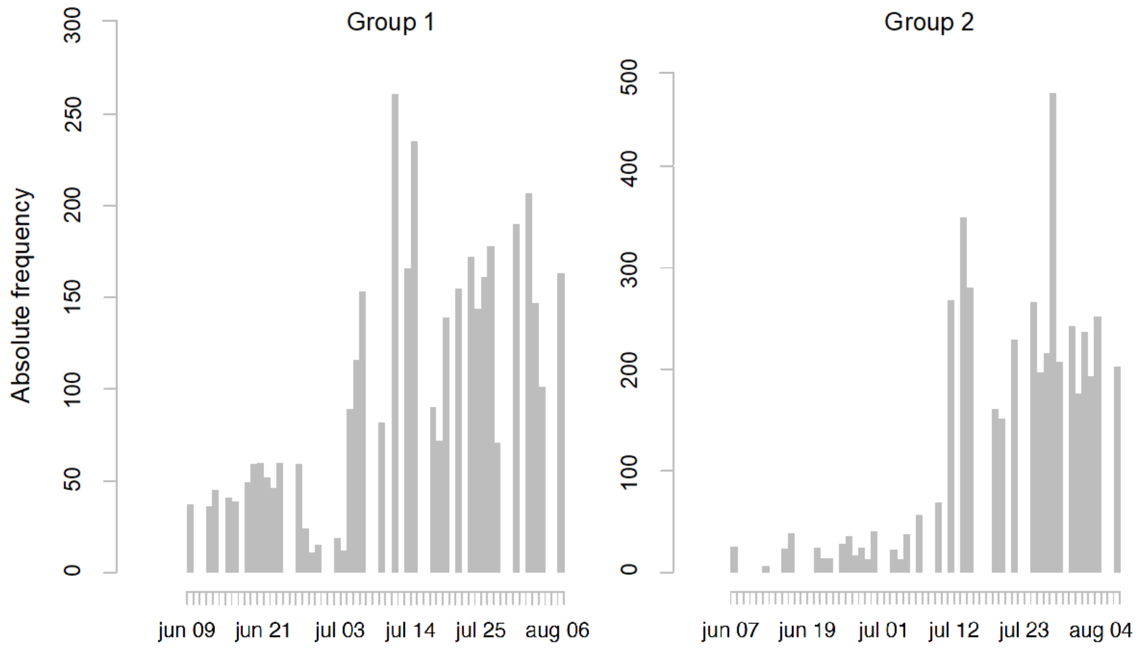

Figure S1. **The within-group demonstrations by the conspecific models.** The frequency by which the trained chimpanzees (one adult female in each group) successfully used the apparatus (*y*-axis) over experimental time (*x*-axis) for Group 1 (left) and Group 2 (right).

Table S5. Subject details including age, sex, mother identity, origin, and whether the subject participated in the experiment.

Chimpanzee	*Group*	*Age*[*]	*Sex*	*Mother*	*Origin*	*Participated in exp.*[**]
Pal	1	36.5	male	unknown	wild born	yes
Booboo	1	35.5	male	unknown	wild born	yes
Girly	1	35.5	female	unknown	wild born	yes
Tobar	1	35.5	male	unknown	wild born	yes
Rita	1	34.5	female	unknown	wild born	yes
Tara	1	34.5	male	unknown	wild born	yes
Ingrid	1	26.5	female	Liza	captive born	yes
Brenda	1	21.8	female	Bella	captive born	yes
Renate	1	20.5	female	Rita	captive born	yes
Genny	1	20.4	female	Girly	captive born	yes
Bob	1	16.2	male	Big Jane	captive born	yes
Gerard	1	15.2	male	Girly	captive born	yes
Ilse	1	15.1	female	Ingrid	captive born	yes
Regina	1	11.5	female	Renate	captive born	yes
Rusty	1	10.7	male	Rita	captive born	yes
Chrissy	1	10.5	female	Cleo	captive born	yes
Innocentia	1	10.4	female	Ingrid	captive born	yes
Gonzaga	1	9.5	male	Genny	captive born	yes
Irene	1	5.4	female	Ingrid	captive born	yes
Rachel	1	4.8	female	Renate	captive born	yes
Ian	1	2.5	male	Ilse	captive born	no
Gloria	1	1.5	female	Genny	captive born	no
Ida	1	1.3	female	Ingrid	captive born	no
Noel	2	40.5	female	unknown	wild born	yes
Donna	2	33.5	female	unknown	wild born	yes
Coco	2	32.5	female	unknown	wild born	yes
Jane	2	32.5	female	unknown	wild born	yes
Maggie	2	31.5	female	unknown	wild born	yes
Misha	2	29.5	female	unknown	wild born	yes
Dora	2	28.4	female	unknown	wild born	yes
Pan	2	28.4	male	unknown	wild born	yes
Pippa	2	28.4	female	unknown	wild born	yes
Trixie	2	27.4	female	unknown	wild born	yes
Zsabu	2	27.4	male	unknown	wild born	yes
Diana	2	26.4	female	unknown	wild born	yes
Masya	2	26.4	female	unknown	wild born	yes
Violet	2	26.4	female	unknown	wild born	yes
Judy	2	22.4	female	Jane	captive born	yes

Carol	2	21	female	Coco	captive born	yes
Dolly	2	20.6	female	Dora	captive born	yes
Nikkie	2	19.6	female	Noel	captive born	yes
Mikey	2	19.4	male	unknown	wild born	yes
Tess	2	19.4	female	Tina	captive born	yes
Tilly	2	16.4	female	Trixie	captive born	yes
Maxine	2	16.1	female	Misha	captive born	yes
Debbie	2	15.6	female	Donna	captive born	yes
David	2	15.5	male	Diana	captive born	yes
Claire	2	15.1	female	Coco	captive born	yes
Doug	2	14.4	male	Dora	captive born	yes
Nina	2	14.2	female	Noel	captive born	yes
Vis	2	13.1	male	Violet	captive born	yes
Daisey	2	12.6	female	Diana	captive born	yes
Mary	2	12.2	female	Masya	captive born	yes
John	2	11.4	male	Judy	captive born	yes
Jenkins	2	10.4	female	Jane	captive born	yes
Moyo	2	9.8	male	Maggie	captive born	yes
Dizzy	2	9.5	female	Diana	captive born	yes
Charity	2	9.4	female	Carol	captive born	yes
Max	2	8.8	male	Misha	captive born	yes
Jones	2	6.7	male	Jane	captive born	yes
Jacky	2	5.2	male	Judy	captive born	yes
Martin	2	5.2	male	Misha	captive born	yes
Danny	2	5.1	male	Dora	captive born	yes
Mavis	2	4.5	male	Masya	captive born	no
May	2	4.5	female	Maggie	captive born	yes
Chitalu	2	2.4	female	Claire	captive born	no

* at the start of the study
** whether or not the individual took part in the experimental phase, either by observing, attempting, or successfully operating the apparatus

References

1. van Leeuwen, E. J. C. *et al.* Chimpanzees behave prosocially in a group-specific manner. *Sci Adv* **7**, eabc7982 (2021).
2. Franz, M. & Nunn, C. L. Network-based diffusion analysis: a new method for detecting social learning. *The Royal Society Proceedings. Biological sciences* **276**, 1829–36 (2009).
3. Hobaiter, C., Poisot, T., Zuberbühler, K., Hoppitt, W. & Gruber, T. Social network analysis shows direct evidence for social transmission of tool use in wild chimpanzees. *PLoS Biology* **12**, e1001960 (2014).
4. Hoppitt, W. The conceptual foundations of network-based diffusion analysis: choosing networks and interpreting results. *Philosophical Transactions of the Royal Society B: Biological Sciences* **372**, 20160418 (2017).
5. Hoppitt, W., Boogert, N. J. & Laland, K. N. Detecting social transmission in networks. *Journal of Theoretical Biology* **263**, 544–555 (2010).
6. Hoppitt, W. & Laland, K. N. *Social learning: An introduction to Mechanisms, Methods, and Models* . (Princeton University Press, 2013).
7. Burnham, K. P. & Anderson, D. R. *Model Selection and Multimodel Inference: A Practical Information-Theoretic Approach*. (Springer, 2002).
8. Allen, J., Weinrich, M., Hoppitt, W. & Rendell, L. Network-based diffusion analysis reveals cultural transmission of lobtail feeding in humpback whales. *Science* **340**, 485–488 (2013).
9. Atton, N., Hoppitt, W., Webster, M. M., Galef, B. G. & Laland, K. N. Information flow through three spine stickleback networks without social transmission. *Proceedings of the Royal Society B: Biological Sciences* **279**, 4272–4278 (2012).
